# Bioluminescent Microbial Bioreporters: A Personal Perspective

**DOI:** 10.3390/bios15020111

**Published:** 2025-02-14

**Authors:** Shimshon Belkin

**Affiliations:** Institute of Life Sciences, The Hebrew University of Jerusalem, Jerusalem 9190401, Israel; sb@mail.huji.ac.il

**Keywords:** bioluminescence, bioreporters, whole-cell biosensors, toxicity, genotoxicity, environmental monitoring

## Abstract

This review attempts to summarize my three decades-long involvement in, and contribution to, the design, construction and testing of bioluminescent microbial sensor strains (bioreporters). With the understanding that such a document cannot be completely free of bias, the review focuses on studies from my own lab only, with almost no coverage of the parallel progress made by others in similar fields. This admittedly subjective approach by no way detracts from the achievements of countless excellent researchers who are not mentioned here, and whose contributions to the field are at least as important as that of my own. The review covers basic aspects of microbial sensor design, and then progresses to describe approaches to performance improvement of sensor strains aimed at the detection of either specific chemicals, groups of chemicals sharing similar characteristics, or global effects, such as toxicity and genotoxicity. The need for integration of live sensor cells into a compatible hardware platform is highlighted, as is the importance of long-term maintenance of the cells’ viability and activity. The use of multi-member sensors’ panels is presented as a means for enhancing the detection spectrum and sample “fingerprinting”, along with a list of different purposes to which such sensors have been put to use.

## 1. Introduction

Trying to summarize one’s academic career in writing is awkward; it is even more so, as is the case here, when your career has not really ended. I still maintain a dynamic research group, and plan to continue to be active in years to come. The suggestion that I review my long-term involvement in the study of bioluminescent bioreporters, while highly flattering, has thus caught me by surprise. Nevertheless, I have willingly agreed to do so, hoping that it will allow me to show my appreciation and gratitude to the students, technicians, researchers and collaborators from Israel and abroad who played an essential role in this trip, and without whom none of the research achievements described below could have been possible. The current effort is thus not an unbiased comprehensive review of bioluminescent microbial sensors, but rather of the progress made in this field by my coworkers and myself, presented from my own personal viewpoint.

My first encounter with the world of bioluminescent bacteria was in the late Professor Moshe Shilo’s Hebrew University Marine Microbiology course, held in the 1970s at the Interuniversity Institute for Marine Sciences in Eilat. I carried this fascination with me when years later I was part of the teaching team of the very same course, and for almost 20 years have always included at least one microbial bioluminescence-related project to challenge students with.

It was Prof. Eugene Rosenberg (Tel Aviv University), also a teacher on that course who, over an evening beer in Eilat, mentioned the option of employing bioluminescent bacteria for toxicity assessment, as a solution for my difficulty in assaying the toxicity of the industrial wastewaters of the Ramat Hovav chemical industrial park in southern Israel. He suggested that I discuss this with the most prominent Israeli figure in the field, Prof. Shimon (Moni) Ulitzur (Technion, Israel Institute of Technology). My collaboration with Moni was indeed highly constructive, but the path I ultimately took was different from his: while he advocated assaying the decrease in light emission of a natural luminescent bacterium as a measure of sample toxicity (a “lights off” assay, versions of which were later standardized and commercialized [1]), I ended up deeply involved in the development of “lights on” assays.

That crucial turning point took place during my 1993–1994 Sabbatical with Dr. Bob LaRossa’s group at the DuPont Central Research and Development campus in Wilmington, Delaware. Under Bob’s guidance, I was not only initiated in the art of molecular engineering of bioluminescent sensor strains, but I also came to realize that the rather simplistic tool I was mastering could be useful in many different ways, from elucidating the fate of wastewater bacteria in the marine environment to the construction of sensor systems for either specific chemicals, classes of compounds, or general environmental stress conditions.

This review is focused on the research path that stemmed from that encounter and which I follow till this day; it does not touch upon my decades-long involvement with other significant aspects of my academic interests, mostly revolving around diverse facets of environmental microbiology and microbial ecology.

## 2. The Basic Story: Selecting the Appropriate Sensing Element

**Figure 1:**
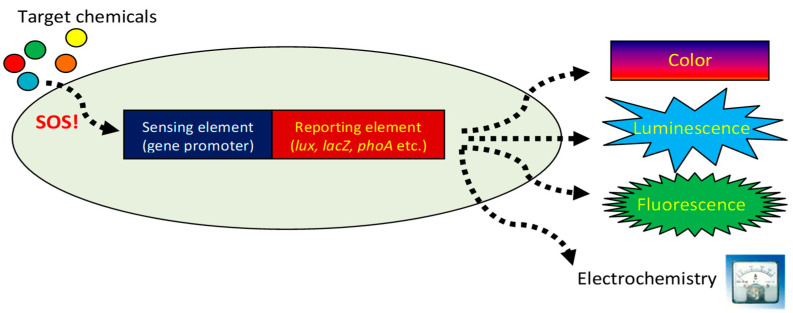
A schematic representation of the basic components of a genetically engineered bioreporter: a sensing element determining the type of chemicals sensed; a reporting element determining the nature of the output signal; and the host cell.

In our hands, the basis for the construction of a microbial sensor strain always involved the a priori selection of three elements (Figure 1): (a) a sensing element (in most cases, a gene promoter induced by the target compound(s) or stress condition); (b) a reporter element—a gene or a group of genes, the activity or the presence of the products of which can be monitored quantitatively; (c) the host microorganism, selected either for convenience or for its relevance to the envisaged application [2,3,4]. Our organism of choice for most applications was the conveniently manipulated *Escherichia coli*. Out of a rather limited list of reporter options—primarily fluorescence, color generation, electric current or bioluminescence—we usually worked with the latter. In addition to the detection sensitivity afforded by the bacterial *luxCDABE(G)* gene cassette, it has the added advantage of intracellular production of the long-chain aldehyde luciferase substrate, alleviating the need for adding it externally, as is the case of other enzymatic reporters. Thus, the main challenge when designing a new sensor strain was the selection of the most suitable sensing element, and—when relevant—identification of its associated regulatory components.

An obvious cornerstone of every scientific progress and innovation is preexisting knowledge. The continuously expanding microbial biochemical/molecular/physiological knowledge base allows the selection of gene promoters that will respond to the presence of many target compounds. This was how, in the pioneering work of Sayler and his colleagues [5], the *nahG* gene promoter was selected as the sensing element in their *Pseudomonas fluorescens* naphthalene sensor, and how shortly afterwards the *merR* system served as an excellent component of the first *E. coli* mercury bioreporter [6]. Similarly, the series of papers that came out of the LaRossa group a few years later were all based on the same principle: the promoter of the catalase *katG* gene, for example, served for the construction of a hydrogen peroxide sensor [7], or the promoter of the *recA* gene for a DNA-damage sensor [8].

However, how do you select a sensing element if there is no straightforward information as to a cellular response to a compound, or to a biochemical reaction involved in its metabolism? The first DNA chips introduced in the early 1990s provided a high-throughput unbiased answer: the promoters of genes, shown on-chip to be induced by the target chemical or condition, may be suitable candidates for sensor construction. In our hands, regrettably, this promising technology did not yield the desirable results; a different approach was much more successful: employing a transcriptional GFP fusion library constructed in the Alon’s lab at the Weizmann institute [9] has identified several *E. coli* gene promotors responsive to 2,4-dinitrotoluene (DNT) [10], the signature chemical accompanying 2,4,6-trinitrotoluene (TNT), the main explosive component of landmines. The most prominent among those was *yqjF*, the gene promoter that served as the sensing element in a subsequent series of DNT/TNT sensor strains. Another relevant DNT-inducible gene promoter, *azoR*, was similarly identified by the use of RNA-Seq analysis [11], another methodology that allows for an unbiased screening for genes responsive to specific environmental conditions.

## 3. How to Improve Your Sensor’s Performance

The basic promoter–reporter fusion, the core concept in microbial bioreporter design, is as effective as it is simple [3]. Often, however, even if the desired specificity is achieved, other performance parameters, most importantly signal intensity, detection sensitivity and response time, are insufficient for the envisaged application. Over the years we have adopted and developed several strategies for performance enhancement. These spanned genetic manipulations of the sensing, reporting and regulatory elements of the original construct, as well as of the host cell.

Host cell modifications mostly included mutations which either affected cellular permeability [12,13] or enhanced sensor performance for reasons that were not always clear. Worth mentioning in the latter category is B. Shemer’s monumental screening effort of the entire *E. coli* mutation spectrum for mutations beneficial to *yqjF* activation by DNT [14]. Two of the mutations identified in that study, in the *ygdD* and *eutE* genes, are still a part of the bioreporter in use today for explosives’ detection.

Beneficial adjustments to the bioluminescence reporter element included either switching the entire *lux* gene cassette, achieving variations in temperature dependence as well as in signal intensity [15], or splitting the *lux* gene cassette into two independently regulated components, *luxAB* and *luxCDE* [16]. The latter strategy, by providing an increased substrate supply to luciferase due to a constitutive expression of *luxCDE*, removed a potential bottleneck in the enzyme’s activity and boosted its light production.

Significant enhancements in DNT detection performance were also achieved by modifying YhaJ, the LysR-type transcriptional regulator of *yqjF* [17]. Two different experimental approaches were adopted for this purpose: “Directed evolution” of the YhaJ protein by random mutagenesis [18], and screening a focused saturation library of over 6 × 10^7^ mutated YhaJ variants [19]. Directed evolution by several consecutive rounds of random mutagenesis was also successfully employed for significant enhancement of the *yqjF* gene promotor [10,20], as well as for “gene shuffling” performed on a fusion of the *yqjF* and *azoR* gene promotors [21]. Further improvements were achieved by the introduction of quorum sensing elements [22] into the induction chain, as well as the modification of specific ribosomal binding sites. Several other manipulations we and others have successfully integrated into microbial bioreporter designs are described elsewhere [23].

## 4. Just Having a Good Sensor Strain May Not Be Enough

The term “Biosensor” is defined by the International Union of Pure and Applied Chemistry [24] as a “device that uses specific biochemical reactions mediated by isolated enzymes, immuno-systems, tissues, organelles or whole cells”. In other words, in order to be considered a “biosensor”, the bioreporter cells need to be integrated into a hardware platform that will allow, for example, field or point-of-care use. Numerous approaches for the integration of live cells into inanimate devices have been described in the scientific literature [25,26]. Our contributions to these efforts were manifested in several directions. Melamed et al. have demonstrated that nanodrops of functional live sensor cells can be spotted onto a glass surface [27] and be successfully revived after storage. For online water monitoring, Sharon Yagur-Kroll and Tal Elad et al. have demonstrated the integration of bioreporter cells in miniature porous aluminum oxide-based [28] and microfluidic polydimethylsiloxane (PDMS)-based flow cells [29], respectively. The latter device uninterruptedly monitored untreated tap water for two weeks. A different approach was adopted by the group of A. J. Agranat, who successfully incorporated alginate-immobilized bioreporters into optoelectronic biosensor modules designed for detection of trace explosives in the soil under the device’s footprint [30]. In the group of Ji-Yen Chen in Academica Sinica, Taiwan, the cells were immobilized in agar-agar and embedded in several biosensor devices, including a smartphone-based variant, which allowed sensitive detection of simulated pollutants in water and antibiotics in foodstuff [31,32,33]. Other original approaches for the integration of live bacterial sensor cells with a signal transducer were demonstrated by the group of R. S. Marks, who successfully immobilized genotoxicity bioreporters onto the tips of optic fibers [34,35], of U. Levy, who immobilized and visualized single reporter cells in nano-plasmonic waveguides [36], and of T. Scheper, who sensitively measures gaseous DNT by placing alginate-immobilized sensor cells in front of a photodiode housed in a 3D-printed environmentally controlled chamber [37].

An essential prerequisite for live cell integration into any kind of hardware platform is ensuring the long-term maintenance of their viability and activity. In most reported cases, this ultimately involves encapsulation in a polymeric matrix; out of several available hydrogel options, we have mostly adhered to either agar-agar [29] or alginate [15]; the Hebrew University group of S. Magdassi and L. Larush has amended the latter with several additives that efficiently slowed dehydration rates [19]. A completely different tactic was proposed and demonstrated by O. Lev (Hebrew University), who immobilized fluorescent sensor cells in bio-compatible sol-gel biofilms [38,39,40,41]. Though possibly less significant for integration into biosensor devices, cell viability preservation by lyophilization (freeze drying) is also highly relevant for conservation of bioreporter performance. When carried out properly, removal of water by sublimation from frozen cells can be highly beneficial for long-term maintenance of cell viability [41,42,43].

## 5. Toxicity and Genotoxicity Assessment, the Panel Approach and Group Classification

Whole-cell sensor cells may be broadly divided into two groups: those designed to detect specific chemicals or groups of related compounds, and those aimed at the detection of global effects, such as toxicity or genotoxicity, for monitoring non-specific toxic threats in media such as water, air or food. The question addressed by sensors from the latter class is not “what are the specific toxic components of the sample?”, as in most chemical analytical techniques, but rather a general “how toxic is the sample?” Following an initial attempt at the use of “lights off” assays [44,45,46] for this purpose, I was attracted by the approach of Robert LaRossa and his group in Dupont, who championed the “lights on” induction of specific *E. coli* gene promoters as an indication of sample toxicity. For the detection of overall toxicity, the gene promoter acting as the sensing element needs to be as non-specific as possible [47,48,49,50]: *recA* for the detection of DNA damage [8], *micF* [51,52] and *katG* [7] for the detection of oxidative stress, *fabA* for membrane damage [53], *grpE* for heat-shock inducing chemicals [51,54], etc. It soon became clear, however, that, regardless of the breadth of its response spectrum, no single reporter strain can detect all potential threats. For that reason, bioreporter panels were proposed, which cover a much more extensive range of toxic chemicals. The advantages of such panels lie not only in their broad coverage; the combined responses to the same sample of different sensors with different specificities not only provide information as to the presence of toxic compounds but also generate a “fingerprint” that may indicate the chemical or biological nature of the sample components. This has been demonstrated using sensor panels composed of seven [55] and five [56] bioreporter strains, a 12-member panel for antibiotics detection [57,58], and much larger panels of fluorescent sensors for chemical [59] and pharmaceutical [60] classification. A printed solid-phase array for heavy metal detection was described by Chien et al. [61], and for antibiotics detection and characterization by Huang et al. [62]. The array concept has also been covered in several reviews [63,64].

Another sub-class of microbial sensors are those for which the specificity is directed not at single chemicals but to groups of chemicals sharing similar chemical structures or biological activities. Examples include heavy metals [65,66], halo-organic acids [67], chloro-catechols (in combination with heavy metals) [68], as well as the yeast-based sensors of endocrine-disrupting chemicals described in Section 7 below.

## 6. Detection of Specific Compounds and Buried Explosives

As noted above, microbial sensors may be genetically engineered and employed to detect and respond to specific compounds. Such was the detection of p-chloro-benzoic acid described by Y. Rozen and S. Kohler [69,70], of 1,3,5-trinitro-1,3,5-triazinane (RDX) by Lifshitz et al. [71], and of methyl (1R,2R,3S,5S)-3- (benzoyloxy)-8-methyl-8-azabicyclo [3.2.1] octane-2-carboxylate (cocaine) by Grimm et al. [72].

**Figure 2:**
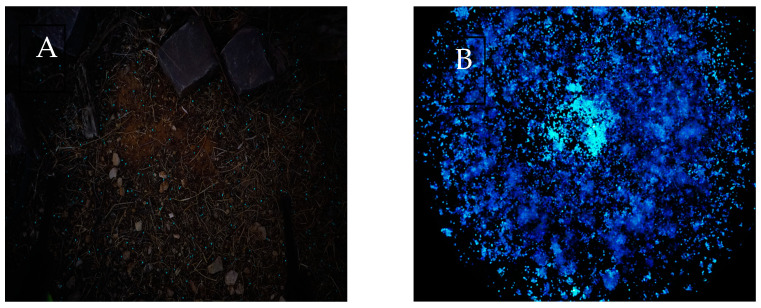
DNT-sensing bioreporters encapsulated in alginate beads spread over an uneven test field (**A**) and over a container harboring an anti-personnel mine buried in sand ((**B**), bar = 5 cm). Images taken at night with a Sony α7SII camera equipped with a Sony FE 28 mm f/2 lens (F = 2, ISO = 5000, Exposure = 6 s). Image in panel A is by Etai Shpigel, and in panel B is from [15].

Probably the longest running single research effort in our group involves the design, construction and testing of microbial sensor strains for the remote detection of buried explosives. While the concept had already been proposed over 25 years ago and has been awarded at least two patents [73,74], there was no evident follow up, and no scientific article on the topic has been published. We have first addressed this challenge in 2014, when we first described *E. coli* strains capable of sensing trace amounts of 2,4,6 trinitrotoluene (TNT) and 2,4 dinitro-toluene (DNT) [10]. Since then, we have reported progressively improved explosive detection performance by newer bioreporter generations [14,15,18,19,20,21,22,75,76]. Figure 2A displays a night image of alginate beads harboring bioluminescent DNT-sensing bacteria dispersed on an uneven field, and Figure 2B of similar beads over a buried anti-personnel landmine.

Hopefully the coming years will see an actual application of this emerging technology, playing a role in the endless war against the humanitarian tragedy of unexploded landmines worldwide.

## 7. Not Only Prokaryotes

Traditionally, most whole-cell biosensors harbor bacteria as their sensing entities, mostly due to their facility for growth and maintenance and their easy amenability to molecular manipulations. The field, however, is not exclusively prokaryotic; numerous eukaryotic cell types, from fungi to mammalian, have also been employed in varied sensing applications, from environmental monitoring to medical diagnostics and imaging. A prominent group of eukaryotic microorganisms belonging to this category are yeasts, most often the baker’s yeast *Saccharomyces cerevisiae*. Notable among the genetically engineered yeast sensors are those designed for the detection of traces of endocrine disrupting compounds (EDCs), both estrogenic and androgenic in nature, in environmental samples. Under the leadership of Liat Moscovici, and with close collaboration with Georg Reifferscheid and Sebastian Buchinger from the German Federal Institute of Hydrology, several sensitive fluorescent *S. cerevisiae*-based EDC sensor strains have been developed [13,77]. These were tested not only on model chemicals in liquid samples but also on complex wastewater samples separated on high-precision thin layer chromatographic plates [78]. The latter effect-based approach, which was also successfully demonstrated for the detection and separation of genotoxic agents in wastewater [79], allows to single out the individual components responsible for the sample’s deleterious activity.

## 8. Not Only Pollutants and Explosives

Some of our research efforts involving bioluminescent sensor strains had nothing to do with environmental monitoring. Yael Rozen’s Ph.D. thesis, which revolved around the fate of enteric bacteria released into the marine environment as a result of uncontrolled wastewater discharges, employed luminescent *E. coli* sensor strains to evaluate the nature of the physiological stress exerted by the entry of an intestinal bacterium into the sea [80,81,82]. Michael (Miki) Ionescu has used the same approach while studying the basis of *E. coli*’s reaction to osmotic stress [83,84,85]. Osnat Gillor, in her thesis work, developed bioreporters that assayed the bioavailability of two major essential elements, nitrogen and phosphorus. In the latter case the host microorganism was a cyanobacterium (*Synechococcus* PCC 7942), and the sensor elements were the promoters of the *glnA* [86] and *phoA* [87,88] genes. The reporters were the *Vibrio harveyi luxAB* genes only, necessitating the external addition of decanal as a substrate. These strains were then successfully employed [89] to assess nutrients’ bioavailability to cyanobacterial primary producers in the Sea of Galilee (Lake Kinneret).

Bioluminescent reporter strains were also involved in the study of the antibacterial effects and mode of action of either CdSe quantum dots [90], rice seedlings exudates [91] or UV radiation [92,93], as well as that of particulate air samples [94]. The role of such sensors was also paramount in unravelling the DNA-damaging effects of lyophilization [95], a common method for long-term preservation of microbial cultures, and in the study of the bactericidal action of hypochlorous acid [96].

## 9. Summary

The present review highlights some of the roles my coworkers and I have played, and the contributions we have made, along the road opened in the early 1990s, when the first bioluminescent microbial sensor strains were described [5,6]. That groundbreaking period paved the way for numerous reports describing whole-cell sensor systems of different origins and diverse applications. Now, over 30 years later, the scientific literature abounds with descriptions of cell-based sensors, mostly microbial, with potential applications in diverse fields, including environmental, industrial, security, or medical diagnostics [97,98,99,100]. An obvious question arises from reading these articles: if such sensor systems are so good, why have so few of them been adopted by regulatory and other relevant authorities as part of approved toolkits for relevant applications?

One obvious answer, and the simplest, is that, in many cases, cell-based sensors are simply inferior to chemical analysis. Modern analytical equipment can often provide information regarding a sample’s composition at levels of sensitivity and accuracy that no live bioreporter is capable of. Many of the aforesaid publications, therefore, remain as elegant exercises in molecular biology without the potential for maturing into a real application. Another inhibitory factor may be the complexity and time consumption involved in the successful integration of a new method into an existing set of regulations, or as an integral part of standard analytical or diagnostic practices. The use of naturally luminescent bacteria in toxicity bioassays serves as an excellent example of this point: the time between the first scientific publications in the 1970s and their regulatory adoption in the 1990s [1] was roughly 15–25 years. More specific potential problems are associated with the fact that the functionality of live cells is by nature restricted to a rather limited set of environmental conditions, as well as with the limitations of long-term preservation of cellular viability and activity. In many cases, these cannot compete with the shelf life of chemicals and cell-free reagents. In addition, all of these important concerns are overshadowed by issues associated with the use and environmental release of genetically modified microorganisms. These have important regulatory and public perception aspects which need to be addressed, as recently pointed out by Chemla et al. [101].

These constraints notwithstanding, the advantages of using live sensor cells as analytical tools should nevertheless be highlighted. As indicated in Section 5 above, no chemical analysis or molecule-based biosensor can answer questions such as “how toxic is the sample” or “how mutagenic is a chemical”. Monitoring the responses of a live organism is essential for this purpose, and microbial systems are already replacing toxicity and genotoxicity assays based on higher organisms [102]. In a similar vein, only a live cell can report on the bioavailability of a pollutant, as compared to the chemical’s total concentration in a sample. This information may be important, for example, in assessing the risk in contaminated soils, where the bioavailable components may pose a much higher risk than the fraction tightly adsorbed to soil particles. Another inherent advantage in the use of live bioreporters is the technical possibility of using them in schemes in which the sensing agent (i.e., the live bioreporter) is separated in space from the signal capturing hardware. An example of this type of application is the standoff detection of buried explosives [75], in which bioluminescent DNT/TNT-sensing bacteria are spread on the ground and the imaging apparatus is positioned remotely.

Will these advantages be better utilized in the future? with the increasing introduction of engineering principles into molecular circuits’ design [103,104], along with the envisaged advent of artificial intelligence-based tools, one can hope that the field will continue to evolve and expand, potentially impacting different aspects of our lives.
